# Screening and Counseling Practices Reported by Obstetrician–Gynecologists for Patients With Hepatitis C Virus Infection

**DOI:** 10.1155/S106474490300005X

**Published:** 2003

**Authors:** Kathy Boaz, Anthony E. Fiore, Stephanie J. Schrag, Bernard Gonik, Jay Schulkin

**Affiliations:** 1 National Center for Infectious Diseases Division of Viral Hepatitis Centers for Disease Control and Prevention MS-G37, 1600 Clifton Rd Atlanta GA 30333 USA; 2 Department of Obstetrics and Gynecology Wayne State University School of Medicine Detroit MI USA; 3 American College of Obstetricians and Gynecologists Department of Research Washington DC USA

## Abstract

*Background:* Obstetrician—gynecologists are important providers of primary health care to women, and the hepatitis C virus (HCV) infection screening practices and recommendations provided by obstetrician—gynecologists for HCV-infected patients are unknown.

*Methods:* We surveyed American College of Obstetricians and Gynecologists (ACOG) Fellows, including 413 Fellows who were participating in the Collaborative Ambulatory Research Network (CARN) and 650 randomly sampled Fellows, about HCV screening and counseling practices.

*Results:* In total, 74% of CARN members and 44% of non-CARN members responded. Demographics and practice structure were similar between the two groups. More than 80% of providers routinely collected drug use and blood transfusion histories from their patients. Of the respondents, 49% always screened for HCV infection when patients had a history of injection drug use, and 35% screened all patientswho had received a blood transfusion before 1992. For HCV-infected patients, 47% of the physicians always advised against breastfeeding, 70% recommended condom use with a long-term steady partner, and 64% advised against alcohol consumption. Respondents who considered themselves to be primary care providers were no more likely to screen or provide appropriate counseling messages than were other providers.

*Conclusions:* Most obstetrician—gynecologists are routinely collecting information that can be used to assess HCV infection risk, but HCV screening practices and counseling that are provided for those with HCV infection are not always consistent with current Centers for Disease Control and Prevention and ACOG recommendations.


*Infect Dis Obstet Gynecol 2003;11:39–44*

### Screening and counseling practices reported by obstetrician–gynecologists for patients with hepatitis C virus infection

**Kathy Boaz[1], Anthony E. Fiore[1], Stephanie J. Schrag[1], Bernard Gonik[2] and Jay Schulkin[3]**

[1]*National Center for Infectious Diseases, Centers for Disease Control and Prevention, Atlanta, GA*
[2]*Department of Obstetrics and Gynecology, Wayne State University School of Medicine, Detroit, MI and*
[3]*American College of Obstetricians and Gynecologists, Department of Research, Washington, DC*

***Background:*** Obstetrician–gynecologists are important providers of primary health care to women, and the hepatitis C virus (HCV) infection screening practices and recommendations provided by obstetrician–gynecologists for HCV-infected patients are unknown.
***Methods:*** We surveyed American College of Obstetricians and Gynecologists (ACOG) Fellows, including 413 Fellows who were participating in the Collaborative Ambulatory Research Network (CARN) and 650 randomly sampled Fellows, about HCV screening and counseling practices.
***Results:*** In total, 74% of CARN members and 44% of non-CARN members responded. Demographics and practice structure were similar between the two groups. More than 80% of providers routinely collected drug use and blood transfusion histories from their patients. Of the respondents, 49% always screened for HCV infection when patients had a history of injection drug use, and 35% screened all patients who had received a blood transfusion before 1992. For HCV-infected patients, 47% of the physicians always advised against breastfeeding, 70% recommended condom use with a long-term steady partner, and 64% advised against alcohol consumption. Respondents who considered themselves to be primary care providers were no more likely to screen or provide appropriate counseling messages than were other providers.
***Conclusions:*** Most obstetrician–gynecologists are routinely collecting information that can be used to assess HCV infection risk, but HCV screening practices and counseling that are provided for those with HCV infection are not always consistent with current Centers for Disease Control and Prevention and ACOG recommendations.

Key words: VIRAL TRANSMISSION AND PREGNANCY; VIRAL TRANSMISSION AND BREASTFEEDING; VIRAL TRANSMISSION AND CESAREAN SECTION; PREGNANCY GUIDELINES; OBSTETRICS GUIDELINES

Obstetrician–gynecologists provide more office-based general medical examinations for women aged 15 years or older than either general family practitioners or internists[1], and may be the sole primary care provider for some women. They can provide an important service by assessing hepatitis C virus (HCV) infection risk, testing women with risk factors, and counseling HCV-positive patients.

Correspondence to: Dr Anthony Fiore, Division of Viral Hepatitis, MS-G37, Centers for Disease Control and Prevention, 1600 Clifton Rd, Atlanta, GA 30333, USA. E-mail: afiore@cdc.gov

© 2003 The Parthenon Publishing Group 

The Centers for Disease Control and Prevention (CDC) and the American College of Obstetricians and Gynecologists (ACOG) have both published recommendations for screening individuals for HCV infection and counseling those who are HCV infected. Current CDC recommendations are that physicians in primary care settings should provide HCV screening for patients with risk factors for HCV infection, such as illicit injection drug use or receipt of a transfusion of blood or blood components before July 1992[2]. ACOG has indicated that obstetrician–gynecologists should include screening for HCV infection among these risk factor groups as part of primary and preventive care[3]. Neither ACOG nor CDC has recommended that pregnant women be routinely screened or that HCV-infected women have Cesarean sections and abstain from breastfeeding in order to avoid transmission of the virus to the newborn[2–4]. CDC does not recommend that HCV-infected patients be counseled to change their sexual practices if they have one long-term steady partner[2]. Both CDC and the National Institutes of Health recommend that HCV-infected patients should be counseled to avoid alcohol[2,5].

The extent to which obstetrician–gynecologists have incorporated current HCV screening and counseling recommendations into their practices has not been determined. As part of a larger survey on disease screening practices of a national sample of obstetrician–gynecologists, we included questions to determine how practitioners collect information on HCV risk factors and to assess their HCV screening and counseling practices.

### METHODS

In February 2001, questionnaires were mailed to two groups of Fellows of ACOG. The first group, namely the Collaborative Ambulatory Research Network (CARN), includes 413 ACOG Fellows who have volunteered to participate in surveys; CARN participants have been chosen to reflect the age and gender distribution of all ACOG Fellows. Questionnaires were also sent to a computer-generated random sample of 650 Fellows selected from the ACOG membership list who had not previously been selected to participate in an ACOG survey during the same calendar year. Questions were in either multiple-choice or scaled-response format. A second mailing was sent 1 month after the first to those individuals who did not respond to the first mailing.

The survey included questions about the practitioner, his/her practice, the demographic characteristics of patients, and specific questions about screening and counseling practices for HCV infection. We performed univariate analyses to evaluate factors associated with HCV risk factor assessment, screening and counseling practices, and we performed stratified analyses to examine the effect of practitioner age, location and practice type, as well as practitioner self-description as a primary care provider, on survey responses. Survey responses were analyzed in SAS version 8 (SAS Institute, Cary, NC). We assessed differences for categorical measures using the Chi-square test statistic. A $p$-value of $< 0.05$ was considered to be statistically significant.

### RESULTS

Completed surveys were received from 307 (74%) CARN members and 286 (44%) non-CARN members. Respondents who had not seen obstetric patients in the year 2000 (two CARN respondents and eight non-CARN respondents) were excluded. The characteristics of the CARN and non-CARN groups are summarized in Table 1. The median age of respondents was 48 years in the CARN group and 46 years in the non-CARN group. In both groups, the majority of respondents were male obstetrician–gynecologists (rather than specialists in either obstetrics or gynecology) who practiced in urban or suburban settings. There were no statistically significant differences between CARN and non-CARN members in provider demographic characteristics such as age, sex, race or ethnicity, or in practice characteristics such as location (urban, rural or suburban), type (multispecialty group or not) or proportion of patients receiving Medicaid. However, CARN members were more likely than non-CARN members to consider themselves to be primary care providers (60 vs. 49%; $p = 0.007$). Because of the demographic and practice

**Table 1** Demographic and practice characteristics of survey respondents in the Collaborative Ambulatory Research Network (CARN) and non-CARN groups

| *Characteristic* | *CARN group* (*n* = 305*) | *Non-CARN group* (*n* = 278*) |
|---|---|---|
| *Provider characteristics* | | |
| Median age (years) | 48 | 46 |
| Male sex (%) | 170 (56) | 177 (64) |
| Self-identifies as a primary care provider (%) | 181 (60) | 135 (49)† |
| *Practice characteristics* | | |
| Practice structure | | |
| Obstetrics/gynecology group (%) | 141 (46) | 118 (42) |
| Solo practice | 75 (25) | 64 (23) |
| Other | 87 (29) | 96 (35) |
| Practice location | | |
| Urban (%) | 42 | 44 |
| Suburban (%) | 41 | 41 |
| Rural (%) | 17 | 15 |
| Patients receiving Medicaid (%) | | |
| 0–10% | 38 | 37 |
| 11–40% | 41 | 39 |
| > 40% | 21 | 24 |

*Totals vary for each characteristic due to missing responses; †Pearson $\chi^2$ test: $p < 0.001$

similarities between the CARN and non-CARN respondents, we combined the CARN and non-CARN responses for the analysis.

Most providers (89%) reported collecting medical history information from new obstetric and gynecological patients on standardized forms. Among the providers who used a standardized form, 93% included at least one question that could identify a risk factor for HCV infection (86% requested information on use of injection drugs and 87% resquested information on blood transfusion). Respondents who considered themselves to be primary care providers were no more likely to collect information on drug use ($p = 0.72$) or transfusion history ($p = 0.43$) on standardized forms than were those who did not consider themselves to be primary care providers. Provider characteristics (including age, sex, practice type, and practice location) were not associated with collection of information on risk factors for HCV infection.

**Table 2** Groups screened and approximate frequency of screening for hepatitis C virus infection as reported by providers

| | *Provider recommends screening (%)* | | |
|---|---|---|---|
| *Group screened* | *Always* | *Sometimes* | *Never* |
| Women who have ever injected illegal drugs | 49 | 40 | 11 |
| Women who received a blood transfusion before 1992 | 35 | 46 | 19 |
| Pregnant women | 11 | 56 | 33 |

HCV screening practices are summarized in Table 2. Approximately half of the respondents reported that they performed HCV infection screening on all obstetric and gynecological patients who reported ever having injected illicit drugs, and 35% screened all individuals who reported having received a transfusion before 1992. Physicians who considered themselves to be primary care providers were not more likely to screen patients with a history of illegal drug use ($p = 0.9$) or transfusion ($p = 0.9$) compared with physicians who did not identify themselves as primary care providers (drug use, $p = 0.9$; transfusion, $p = 0.9$). Furthermore, practice type (multispecialty, solo practice or obstetrics/gynecology group) was not associated with frequency of screening for these two risk factors (drug use, $p = 0.31$; transfusion, $p = 0.15$). Physicians whose practices were located in urban or suburban areas were more likely always to screen patients who reported current or past illicit injection drug use than were physicians whose practices were in rural settings ($p = 0.037$). Only 1.5% of respondents reported routine screening for HCV infection of all patients who received gynecology care in their practice. However, 11% reported screening all pregnant patients for HCV infection.

Table 3 summarizes the recommendations and counseling measures for HCV-infected patients reported by those physicians who were surveyed. In total, 6% of providers reported that they always recommended Cesarean sections for pregnant women with HCV infection, and 47% always advised against breastfeeding. A total of 70% of the respondents advised HCV-positive women

**Table 3** Percentage of providers who made specific recommendations to patients with hepatitis C virus infection, according to provider age group

| *Recommendations given to all HCV-infected patients* | *Total (%)* | *Age quartile (%)* | | | |
|---|---|---|---|---|---|
| | | *< 40* | *40–47* | *48–54* | *≥ 55* |
| Cesarean section | 6 | 6 | 8 | 5 | 4 |
| Avoid breastfeeding* | 47 | 55 | 50 | 37 | 43 |
| Use condoms with steady partner* | 70 | 78 | 71 | 65 | 63 |
| Avoid alcohol† | 64 | 67 | 68 | 60 | 56 |

*$\chi^2$ test for trend by age of provider: $p < 0.05$; †$\chi^2$ test for trend by age of provider: $p = 0.053$

to use condoms with a steady partner, and avoidance of alcohol was always recommended by 64% of providers. Advice about breastfeeding, condom use and alcohol consumption varied according to age of the provider and location of the practice. Younger providers were more likely to advise HCV-infected women against breastfeeding ($p = 0.026$) and alcohol consumption ($p = 0.05$) and to recommend condom use with a steady partner ($p = 0.009$). Providers in rural or suburban areas were also more likely to advise HCV-infected women against breastfeeding ($p = 0.001$) and alcohol consumption ($p = 0.05$) and to recommend condom use with a steady partner ($p = 0.001$). Counseling messages did not differ significantly among practitioners in different types of practices.

### DISCUSSION

Obstetrician–gynecologists have a key role in providing primary care to women, and over half of the ACOG members who responded to our survey considered themselves to be primary care providers. On the basis of our survey, some obstetrician–gynecologists do not appear to be consistently providing HCV screening for their patients with the most common risk factors for HCV infection in the USA. When HCV-positive women are identified, some obstetrician–gynecologists are providing counseling messages that differ from the CDC and ACOG recommendations.

Interpretations of these findings are subject to several limitations. We were unable to compare the demographic characteristics of survey respondents with those of nonrespondents, and the screening and counseling practices reported by survey respondents were not confirmed by medical record reviews. In addition, the survey question format did not provide an opportunity for practitioners to indicate that they always refer patients with risk factors or HCV infection to another physician, and therefore do not screen for or counsel patients about HCV infection. However, the survey findings are consistent with surveys of other physician specialties. In one recently published national survey of internists and family physicians, 70% of respondents reported that they always perform HCV screening for patients with risk factors[6]. Finally, due to survey length limitations, we did not determine whether the differences between survey respondents' practices and CDC or ACOG recommendations were due to lack of knowledge or to disagreement with the recommendations. A follow-up survey may be necessary to identify the reasons why HCV-related practice guidelines are not followed by some obstetrician–gynecologists.

HCV infection is the most common chronic bloodborne infection in the USA, with an estimated 2.7 million individuals infected, including approximately 900 000 women[7]. The identification of HCV-infected women is important to enable appropriate medical referral, treatment and risk-reduction counseling to be offered. Current and past use of injected illegal drugs and receipt of a blood transfusion or blood products before July 1992 are among the most important risk factors for HCV infection in the USA. An estimated 60% of new HCV infections are attributable to injection of illegal drugs, and an estimated 79% of current injection drug users have HCV infection[2]. More than 80% of the survey respondents collected patient information on injection of illegal drugs and transfusion history that could be used to assess the HCV infection risk, but less than half reported routine testing of all patients with these risk factors. Some practitioners tested all pregnant women (a low-risk group in most settings), while many others did not test women with risk factors. These results indicate that obstetrician–gynecologists may benefit from education about the rationale for the recommended strategies for HCV infection screening.

The risk of HCV transmission from mother to infant during the pre- and perinatal period is estimated to be approximately 4–6%[2,8–10]. The only factors that have consistently been shown to increase the risk of transmission are the presence of HCV RNA in maternal blood at the time of birth and HCV/HIV coinfection[2,11,12]. Most studies that have assessed whether mode of delivery influences the risk of HCV transmission to a newborn born to an HCV-positive, HIV-negative woman have found that mode of delivery is not an important risk factor for perinatal transmission[11–14]. A randomized controlled trial to evaluate the impact of mode of delivery on the risk of perinatal transmission has not been performed. Few of the surveyed physicians in our study reported that they screened all pregnant women or recommended Cesarean sections for all HCV-infected women.

HCV can be found in breast milk[15], but no instance of transmission via breast milk has been documented. Most studies, including a recent meta-analysis, have not found breastfeeding to be a risk factor for perinatal transmission[10–13,15,16]. CDC recommends that HCV-infected women with cracked or bleeding nipples should consider abstaining from breastfeeding, but there is no general contraindication to breastfeeding[2]. In addition, a recent ACOG committee opinion stated that current evidence indicates breastfeeding does not appreciably increase the risk of transmission of HCV[4]. However, almost half of the survey respondents always advised HCV-infected mothers to avoid breastfeeding their infants.

The majority of the survey respondents recommended that HCV-infected women should use condoms with their steady partners. Sexual transmission of HCV infection from one long-term, steady sexual partner to the other sometimes occurs, but at low frequency. In most studies, the prevalence of HCV infection is low among long-term partners of HCV-infected individuals, unless the partner has other risk factors for HCV infection[2,17–19]. Rather than advising routine condom use, the available data indicate that health care providers should discuss this low level of risk with HCV-infected patients, noting that current recommendations do not advise patients to change their sexual practices if they are in a long-term steady sexual relationship[2].

On the other hand, 36% of the respondents did not always counsel their HCV-positive patients about limiting alcohol intake. Alcohol consumption contributes to the progression of liver disease, and clinicians should advise HCV-infected patients to reduce alcohol intake as part of the medical management of HCV infection[5].

For diseases associated with routine gynecological care, obstetrician–gynecologists deliver preventive medical services effectively[1]. However, in their role as primary care providers for women, obstetrician–gynecologists may also need to provide screening and counseling services for non-gynecologic medical conditions such as HCV infection. In addition to the various HCV-related ACOG technical bulletins that have already been published, alternative educational strategies are necessary to integrate prevention counseling into obstetrician–gynecologists' general practices effectively.

### ACKNOWLEDGEMENTS

This study was funded in part by grant MC-00105 from the United States Department of Health and Human Services, Bureau of Maternal and Child Health. The authors would like to thank Miriam Alter, PhD, Laura Riley, MD, and Anne Schuchat, MD, for their thoughtful reviews of this analysis and manuscript.

### REFERENCES

1. Leader S, Perales PJ. Provision of primary preventive health care services by obstetrician–gynecologists. *Obstet Gynecol* 1995;85:391–5
2. Centers for Disease Control and Prevention. Recommendations for prevention and control of hepatitis C virus (HCV) infection and HCV-related chronic disease. *MMWR* 1998; 47(RR-19):1–39
3. American College of Obstetricians and Gynecologists. *Primary and Preventive Care: Periodic Assessments.* ACOG Technical Bulletin No. 246. Washington, DC: American College of Obstetricians and Gynecologists, 2000:1–7
4. American College of Obstetricians and Gynecologists. *Breastfeeding: Maternal and Infant Aspects.* ACOG Technical Bulletin No. 258. Washington, DC: American College of Obstetricians and Gynecologists, 2000:1–16
5. National Institutes of Health. *Management of Hepatitis C: 2002.* NIH Consensus Statements [online] June 2002 (cited January 31, 2003); 19(1). Available from: URL: http://consensus.nih.gov/cons/116/ 116cdc_intro.htm
6. Shehab TM, Sonnad SS, Lok AS. Management of hepatitis C patients by primary care physicians in the USA: results of a national survey. *J Viral Hepat* 2001;8:377–83
7. Alter MJ, Kruszon-Moran D, Nainan OV, *et al.* The prevalence of hepatitis C virus infection in the United States, 1988 through 1994. *N Engl J Med* 1999;341:556–62
8. Schwimmer JB, Balistreri WF. Transmission, natural history, and treatment of hepatitis C virus infection in the pediatric population. *Semin Liver Dis* 2000;20:37–46
9. Wasley A, Alter MJ. Epidemiology of hepatitis C: geographic differences and temporal trends. *Semin Liver Dis* 2000;20:1–16
10. Yeung LTF, King SM, Roberts EA. Mother-to-infant transmission of hepatitis C virus. *Hepatology* 2001;34:223–9
11. Conte D, Fraquelli M, Prati D, *et al.* Prevalence and clinical course of chronic hepatitis C virus (HCV) infection and rate of HCV vertical transmission in a cohort of 15 250 pregnant women. *Hepatology* 2000;31:751–5
12. Resti M, Azzari C, Mannelli F, *et al.* Mother-to-child transmission of hepatitis C virus: prospective study of risk factors and timing of infection in children born to women seronegative for HIV-I. *BMJ* 1998;317:437–40
13. Resti M, Azzari C, Galli L, *et al.* Maternal drug use is a pre-eminent risk factor for mother-to-child hepatitis C virus transmission: results from a multicenter study of 1372 mother–infant pairs. *J Infect Dis* 2002;185:567–72
14. Thomas DL, Villano SA, Riester KA, *et al.* Perinatal transmission of hepatitis C virus from human immunodeficiency virus type 1-infected mothers. *J Infect Dis* 1998;177:1480–8
15. Lin H, Kao J, Hsu H, *et al.* Absence of infection in breast-fed infants born to hepatitis C virus-infected mothers. *J Pediatr* 1995;126:589–91
16. Granovsky MO, Minkoff HL, Tess BH, *et al.* Hepatitis C virus infection in the mothers and infants cohort study. *Pediatrics* 1998;102:355–9
17. Brettler DB, Mannucci PM, Gringeri A, *et al.* The low risk of hepatitis C virus transmission among sexual partners of hepatitis C-infected hemophilic males: an international, multicenter study. *Blood* 1992;80:540–3
18. Everhart JE, Di Bisceglie AM, Murray LM, *et al.* Risk for non-A, non-B (type C) hepatitis through sexual or household contact with chronic carriers. *Ann Intern Med* 1990;112:544–5
19. Zylberberg H, Thiers V, Lagorce D, *et al.* Epidemiological and virological analysis of couples infected with hepatitis C virus. *Gut* 1999;45: 112–16

RECEIVED 07/01/02; ACCEPTED 10/17/02
